# Low incidence of severe acute and chronic graft-versus-host disease in a long-term retrospective study with ATG Grafalon routine use

**DOI:** 10.1007/s00277-023-05479-w

**Published:** 2023-10-03

**Authors:** Flora Kovacsova, Frantisek Folber, Barbora Weinbergerova, Radka Stepanova, Tomas Kabut, Miroslav Tomiska, Marta Krejci, Jiri Mayer

**Affiliations:** 1 Department of Internal Medicine, Hematology and Oncology, University Hospital Brno, Jihlavska 20, 62500 Brno, Czech Republic; 2 School of Medicine, Masaryk University, Brno, Czech Republic

**Keywords:** Acute and chronic GVHD, AlloHSCT, ATG Grafalon

## Abstract

Since 2006, combined graft-versus-host disease (GVHD) prophylaxis with ATG Grafalon has been our department’s base of peri-transplant supportive care. This recent retrospective study included 398 patients who underwent their first allogeneic hematopoietic stem cell transplantation after receiving a defined dose of ATG Grafalon. Our observations recorded reduced incidence of severe acute and chronic GVHD without negative impact on overall survival in a nonselected group with standard and uniform GVHD prophylaxis.

## Introduction

Anti-T-lymphocyte immunoglobulin (ATG Grafalon) is a mix of polyclonal immunoglobulins isolated from rabbit serum immunized with human T-lymphoblasts from the human Jurkat cell line. It is used to reduce the risk of graft rejection and graft-versus-host disease (GVHD) in the setting of an allogeneic hematopoietic stem cell transplantation (alloHSCT) [1]. Our publication illustrates real-world data of defined ATG type and dose in GVHD prophylaxis routine use, still controversial concerning recommendations of GVHD prevention, and long-term outcomes [2].

## Methods

Our retrospective study included all consecutive patients who underwent their first alloHSCT at our department between 2006 and 2020, receiving a total ATG dose of 30 or 60 mg/kg (depending on donor type; sibling or unrelated donor) on days − 3 to − 1 or − 4 to − 2 (with FLAMSA conditioning) together with calcineurin inhibitor and methotrexate or mycophenolate mofetil. Eighty-four patients not receiving ATG for heterogeneous reasons (e.g., non-malignant diseases, bone marrow stem cell source or rarely used conditioning regimens) were excluded. We analyzed (1) the cumulative incidence (CI) and severity of acute (100-day CI) and chronic GVHD (24-month CI) according to Przepiorka grading system, (2) differences depending on human leukocyte antigen (HLA) matching and conditioning regimen intensity, and (3) overall survival (OS). Risk factors influencing the development of acute or chronic GVHD were analyzed by multivariate logistic regression analysis.

## Results

**Fig. 1:**
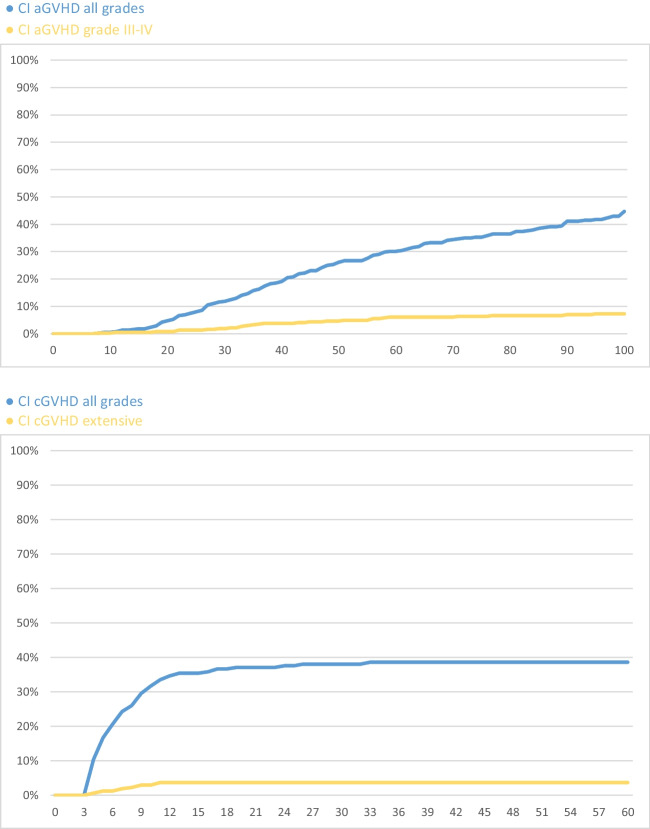
CI aGVHD all grades

Our analysis involved 398 patients: 203 with acute myeloid leukemia (AML) or myelodysplastic syndrome (MDS), 69 with acute lymphoblastic leukemia (ALL), 44 with lymphomas, 40 with myeloproliferative neoplasms including chronic myeloid leukemia, 30 with chronic lymphocytic leukemia (CLL), and 12 other diagnoses (mixed-phenotype acute leukemia, hairy cell leukemia). Myeloablative conditioning was administered to 27.6% (mainly TBI10/Cy and CyBu) and reduced intensity (RIC) in 72.4% of cases (mainly FLAMSA). One hundred seventeen patients (29%) received a graft from a matched sibling donor (MSD), 281 (71%) from an unrelated donor (MUD), 169 of them from a fully matched donor (10 out of 10), 76 with one HLA mismatch (9 out of 10). Patient characteristics are summarized in Table 1. Acute GVHD cumulative incidence at day 100 was 44.7%; severe acute GVHD incidence was 7.3% (Fig. 1). Acute GVHD occurred less often in related donor transplantation compared to full match unrelated donor transplantation (34.8% vs. 45.1%, *p*<0.05) or MUD with one HLA mismatch (53%). Acute GVHD incidence was increased among patients who received a reduced intensity regimen compared to the subgroup with myeloablative conditioning (48.9% vs. 34%, *p*<0.02). These results were confirmed also by the multivariate analysis of acute GVHD risk factors. The odds ratio that a patient who had MUD HSCT will develop acute GVHD was 1.67 compared to MSD. If administering RIC regimen, odds ratio of acute GVHD compared to a MAC regimen was 1.72.

**Table 1:** Patient characteristics

	Number of patients (%)
Total	398 (100%)
Male	221 (55%)
Female	177 (45%)
Diagnosis	
AML/MDS	203 (51%)
ALL	69 (17%)
NHL/HL	44 (11%) 40 (10%)
MPN/CML	30 (8%)
CLL	12 (3%)
Other	
CR at time of HSCT	127 (63%)
AML/MDS	60 (87%)
ALL	27 (61%)
NHL/HL	11 (28%)
MPN/CML	
Conditioning regimen	
MAC:	110 (28%)
TBI10/Cy	56 (51%)
CyBu	42 (38%)
FluBu4	7 (6%)
TBI8/Flu	5 (5%)
RIC:	288 (72%)
FLAMSA	250 (87%)
FluBu2	25 (9%)
TBI2-4/Flu	9 (3%)
Flu/Cy; FluMel	4 (1%)
Donor type	
MUD 9/10	76 (19%)
MUD 10/10	169 (42%)
HLA identical	117 (29%)

*AML/MDS* acute myeloid leukemia/myelodysplastic syndrome, *ALL* acute lymphoblastic leukemia, *NHL/HL* Non-Hodgkin lymphoma/Hodgkin lymphoma, *MPN/CML* myeloproliferative neoplasms/chronic myeloid leukemia, *CLL* chronic lymphocytic leukemia, *CR* complete remission, *MAC* myeloablative conditioning, *TBI10/Cy* 10Gy total body irradiation + cyclophosphamide, *CyBu* cyclophosphamide + busulfan, *FluBu4* fludarabine + busulfan 4 days, *TBI8/Flu* 8Gy total body irradiation + fludarabine, *RIC* reduced intensity conditioning, *FLAMSA* fludarabine + amsacrine + cytarabine + 4Gy total body irradiation + cyclophosphamide, *FluBu2* fludarabine + busulfan 2 days, *TBI2-4/Flu* 2 or 4Gy total body irradiation + fludarabine, *FluCy* fludarabine + cyclophosphamide, *FluMel* fludarabine + melphalan, *MUD* matched unrelated donor, *HLA id* human leukocyte antigen identical

Chronic GVHD cumulative incidence at month 24 was 37.6%; extensive chronic GVHD incidence was 3.7%. As with acute GVHD cases, chronic GVHD occurred less often in related donor transplantation when compared with a full match unrelated donor transplantation (30.6% vs. 34.9%, *p*<0.05) or MUD with one HLA mismatch (44.6%). Chronic GVHD incidence was increased among patients who received a reduced intensity regimen compared to patients with myeloablative conditioning (42% vs. 26%, *p*<0.01).

With univariate analysis we revealed six potential risk factors influencing the development of acute or chronic GVHD including donor gender, donor – patient gender disparity, RIC and MAC conditioning, TBI, donor type, and HLA matching. However, the multivariate analysis verified that only RIC regimen and unrelated donors had effect on the incidence of acute or chronic GVHD. The occurence of chronic GVHD was affected by the history of acute GVHD, with odds ratio of 2.58.

Acute and chronic GVHD cumulative incidences are recorded in Table 2.

**Table 2:** Acute and chronic GVHD cumulative incidence (CI)

CI aGVHD at day 100	All grades	*P* values	Grade III–IV	CI cGVHD at month24	All grades	*P* values	Extensive
Total	44.7%		7.3%	Total	37.6%		3.7%
MUD 9/10	53.0%	0.25	8.6%	MUD 9/10	44.6%	0.16	5.0%
MUD 10/10	45.1%		6.7%	MUD 10/10	34.9%		3.3%
HLA id	34.8%	0.02	6.6%	HLA id	30.6%	0.24	3.1%
MUD total	49.0%		7.6%	MUD total	40.5%		3.9%
RIC	48.9%	0.02	7.8%	RIC	42.0%	0.03	3.7%
MAC	34.0%		5.9%	MAC	26.0%		3.6%

*aGVHD* acute graft-versus-host disease, *cGVHD* chronic graft-versus-host disease, *MUD* matched unrelated donor, *RIC* reduced intensity conditioning, *MAC* myeloablative conditioning

Five-year OS in the whole cohort was 55%. OS was improved when acute or chronic GVHD was present compared to OS without GVHD (OS 57% with aGVHD vs. 53% without aGVHD, *p<0*.02; OS 71% with cGVHD vs. 62% without cGVHD, *p*<0.01). However, the 5-year OS in case of acute GVHD grade III–IV was lower than in the group with acute GVHD grade I–II (37% vs. 61%, *p*<0.01). Ninety-five patients (24%) died due to relapse and 81 for other reasons, resulting in nonrelapse mortality of 20%.

## Discussion

We compare our results with a randomized study by Finke et al., where 201 patients were randomized into groups with dual imunosuppression (methotrexate and calcineurin inhibitor) with our without ATG [3]. This study’s ATG group had a similar incidence of acute GVHD gr. III–IV compared with our patients (11.7%), albeit a higher incidence of extensive chronic GVHD (12.2%).

According to our study, significantly lower incidence of acute and chronic GVHD was present in related donor HSCT. Remberger et al. analyzed the incidence of GVHD in related donor HSCT without ATG and the incidence of GVHD in MUD with ATG prophylaxis. In this study 124 patients were treated with ATG and had MUD HSCT; another 45 patients had MSD HSCT without ATG. They reported significantly lower incidence of acute GVHD grade III–IV (8% vs. 25%) along with extensive cGVHD (5% vs. 40%) in the MUD group with ATG. Based on these results, authors discuss benefit of using ATG even in a related donor setting [4].

Relative to results of several previous studies, reduced intensity conditioning had a controverse impact on GVHD. Afram et al. identified RIC regimens as one of the risk factors for cGVHD development, owing to the persistence of host-origin antigen-presenting cells [5]. Within our cohort, patients with RIC had a higher incidence of both acute and chronic GVHD.

ATG usage in GVHD prophylaxis is detailed in many publications. A metanalysis by Kumar et al. reviews ATG prophylaxis in eight studies [6]. ATG Grafalon was used in three of them. Conclusions of this metaanalysis indicated reduced incidence of acute GVHD grade II–IV and grade III–IV when using Grafalon and decreased incidence of chronic GVHD with both Grafalon and Thymoglobuline. ATG use had no impact on either the OS or non-relapse mortality, yet GVHD-free, relapse-free survival (GRFS) was longer.

Long-term outcomes of ATG in GVHD prophylaxis were confirmed in several additional studies. Finke et al. and Walker et al. showed that cumulative incidence of relapse, NRM, and OS were comparable in groups with or without ATG [7, 8]. Adverse events associated with ATG such as infections did not affect long-term outcomes, although incidence of extensive cGVHD and GRFS was always significantly improved in ATG groups. In the study by Finke, the OS was 49% after 8 years, which is in accordance with our study results (5y OS 55%) [7]. In the study by Walker, the OS was 70%, yet follow-up was only 2 years [8].

Patients with mild forms of GVHD achieved better overall survival probably because of the graft-versus-leukemia (GVL) effect as documented by Weisdorf et al. [9] or Bhatt et al. [10], where mild forms of chronic GVHD decreased disease recurrences resulting in longer OS, despite higher non-relapse mortality.

## Conclusion

Within the potential risk factors of GVHD development we confirmed by this patient cohort analysis that unrelated donors and reduced intensity conditioning regimen were the main factors that increased risk of acute and chronic GVHD incidence. Chronic GVHD also was increased in case of previous acute GVHD.

In conclusion, systematic prophylaxis with ATG Grafalon reduced predominantly severe forms of acute and chronic GVHD both in related and unrelated alloHSCT. We can compare our result of OS with former publications, but other factors that contribute to OS are beyond the scope of our analysis and require further studies to discuss long-term outcomes of using ATG in GVHD prophylaxis.

## Data Availability

The datasets analyzed during the current study are available from the corresponding author on reasonable request.
